# Neutrophil–lymphocyte ratio and subclinical atherosclerosis in essential hypertensive patients

**DOI:** 10.3389/fcvm.2025.1579930

**Published:** 2025-10-31

**Authors:** Luigi Petramala, Francesco Circosta, Leonardo Cremonesi, Danilo Menichelli, Antonino Cimò, Adriana Servello, Emanuela Anastasi, Luca Marino, Claudio Letizia

**Affiliations:** ^1^ Department of Translational and Precision Medicine, “Sapienza” University of Rome, Rome, Italy; ^2^ Department of Medical and Cardiovascular Sciences, “Sapienza” University of Rome, Rome, Italy; ^3^ Department of Experimental Medicine, “Sapienza” University of Rome, Rome, Italy; ^4^ Department of Mechanical and Aerospace Engineering, “Sapienza” University of Rome, Rome, Italy

**Keywords:** Neutrophil–lymphocyte ratio, essential arterial hypertension, subclinical organ damage, immune system, organ damage

## Abstract

**Objectives:**

Arterial hypertension plays a significant role in promoting organ damage and the development of atherosclerosis. The neutrophil–lymphocyte ratio (NLR) is an accessible and cost-effective biomarker that has been strongly associated with adverse outcomes in patients with coronary artery disease and chronic heart failure. The aim of this study was to evaluate the clinical utility of NLR as a surrogate biomarker of subclinical atherosclerotic damage in patients with essential hypertension.

**Methods:**

From January 2024 to November 2024, we consecutively enrolled 346 patients with essential hypertension. For all patients, we collected medical history, anthropometric data, biochemical analyses, and subclinical organ damage, including 24-h urinary excretion of microalbuminuria, carotid intima–media thickness, and transthoracic echocardiography. We excluded patients with arterial hypertension, coronary artery disease, or cerebrovascular or peripheral artery disease.

**Results:**

In our study, we found that patients with higher NLR were associated with high blood pressure values, the use of more than three antihypertensive medications, and a higher prevalence of dyslipidemia and obstructive sleep apnea syndrome. Moreover, elevated NLR values correlated with a higher prevalence of subclinical organ damage (left cardiac ventricular mass, carotid atherosclerosis, and increased microalbuminuria).

**Conclusions:**

Our study shows that in patients with essential hypertension, NLR is significantly correlated with some cardiovascular comorbidities and subclinical organ damage.

## Introduction

Cardiovascular diseases (CVDs), including coronary artery disease (CAD), heart failure, and stroke, remain the leading causes of morbidity and mortality worldwide. In this context, arterial hypertension plays a significant role in promoting the development of atherosclerosis and CVDs (1–3). Arterial hypertension strongly drives the progressive inflammatory processes underlying atherosclerosis, interacting with both innate and adaptive immunity at the vascular level and in target organs (4, 5).

While traditional inflammatory markers such as C-reactive protein (CRP) and IL-6 have been extensively studied, more accessible and cost-effective biomarkers like the neutrophil–lymphocyte ratio (NLR) have gained increasing attention (6). NLR, calculated from routine blood counts, provides a simple measure of systemic inflammation. Neutrophils and lymphocytes are key components of the immune response (7). Neutrophils are primarily involved in acute inflammation and are often elevated in response to infection, stress, or tissue injury. In contrast, lymphocytes represent the adaptive immune response and tend to decrease under chronic stress and inflammatory conditions. Thus, the NLR reflects the balance between these two immune cell populations. A high NLR suggests a shift toward pro-inflammatory processes, which may contribute to endothelial dysfunction and arterial plaque formation, promoting the progression of atherosclerosis (8).

An elevated NLR has been associated with poor outcomes in various CVDs, representing a potential tool for risk stratification (9). Numerous studies have confirmed a strong association between elevated NLR and adverse outcomes in patients with CAD. A high NLR has been linked to greater severity of coronary artery stenosis, worse outcomes following percutaneous coronary intervention, and an increased risk of major adverse cardiovascular events, including myocardial infarction and death (10). A recent meta-analysis highlighted that patients with higher NLRs are more likely to experience recurrent cardiovascular events after acute coronary syndrome (ACS), as well as increased mortality and hospitalization rates related to acute and chronic heart failure (11). The inflammatory environment in heart failure, driven by immune dysregulation and oxidative stress, may be reflected by elevated NLRs, making it a valuable marker of disease. Similarly, in patients with peripheral artery disease (PAD), a high NLR has been correlated with poor outcomes. The chronic inflammatory state in PAD, characterized by vascular inflammation and immune cell activation, is reflected in increased NLRs, suggesting its potential utility in predicting disease progression and related complications (12).

However, limited data are available in the literature regarding the usefulness of NLR in patients with essential arterial hypertension, particularly among those without a history of major cardiovascular events.

The aim of the present study was to evaluate the clinical utility of NLR in patients with essential hypertension, particularly in relation to the evaluation of cardiovascular comorbidities and subclinical atherosclerotic damage.

## Methods

From January 2024 to December 2024, 346 patients (186 men and 160 women) affected by essential hypertension were enrolled at the Center of Arterial Hypertension, Policlinico Umberto I Hospital, University Sapienza, Rome, Italy. All patients underwent anthropometric measurements, fasting venous blood sampling, 24-h urine collection, carotid intima–media thickness (cIMT) assessment, and transthoracic echocardiography.

This study was conducted in accordance with the guidelines of the Declaration of Helsinki II and was approved by the local ethical committee. The study design was clearly written in layperson language and provided to all study participants. Written informed consent was obtained from all patients. Clinical data were obtained as part of routine clinical practice and approved by the Local Ethical Committee of the Department of Clinical, Internal, Anesthesiological and Cardiovascular Sciences, “Sapienza” University of Rome, Italy (date of approval: December 19, 2023).

### Anthropometric measurements

Anthropometric data were collected from all participants. Standing height was measured on barefoot to the nearest 0.5 cm. Weight was measured in light clothing using a platform scale, accurate to the nearest 200 g, with the scale standardized to 0 before each use. Waist circumference was measured to the nearest 0.1 cm using a standard tape placed over the abdomen at the narrowest diameter between the costal margin and the iliac crest. Hip circumference was measured to the nearest 0.1 cm using a non-stretchable standard tape. Measurements were taken over light clothing at the level of the greater trochanter (usually representing the widest diameter around the buttocks). For both waist and hip measurements, the tape was kept horizontal and just tight enough to allow the insertion of a little finger beneath it. Two measurements were taken, and the average value was used for this analysis. Body mass index (BMI) was calculated as weight/(height)^2^.

### Blood arterial pressure assessment and essential hypertension definition

Office blood pressure (BP) was measured using a standard aneroid sphygmomanometer after participants had been seated for 5 min. Systolic blood pressure (SBP) was recorded at the first sound on deflation of the cuff (Korotkoff phase I), and diastolic BP (DBP) was recorded at the complete disappearance of Korotkoff sounds (phase V). Essential hypertension was defined as a BP of 140/90 mmHg or more in three consecutive measurements or in patients receiving antihypertensive therapy. Secondary causes of arterial hypertension were excluded after specific evaluation on the basis of clinical, laboratory, hormonal, and imaging examinations (13). Individuals with a clinical history, clinical symptoms, or electrocardiographic, echocardiographic, or angiographic signs of coronary artery disease, heart failure, cardiomyopathies, or valvular or pericardial diseases were excluded. Individuals with a history of cerebrovascular or peripheral artery disease, hepatic disease, or drug abuse were excluded.

### Measurement of carotid intima–media thickness

A Hewlett-Packard Sonor 5500 Ultrasound system (Hewlett-Packard, Andover, Massachusetts, USA), equipped with a 3.11-MHz real-time B-mode scanner, was used for carotid imaging. The right common carotid artery (CCA) was examined with participants turning their heads 45° to the left. High-resolution images were analyzed to calculate the cIMT, defined as the thickness of the vascular intima–media complex, measured at five consecutive regions of the CCA wall spaced every 4–5 mm, beginning near the bifurcation. For each individual, the cIMT value was calculated as the average of five measurements from the left and five from the right carotid artery. The mean common carotid diameter was defined as the distance across the media–adventitia interface from the near to the far wall and was calculated automatically by averaging measurements taken at 0.1 cm intervals over a 1-cm segment.

### Assessment of echocardiography variables

Echocardiography was performed by expert cardiologists using a General Electric Vivid 7 ultrasound machine (General Electric Medical Systems, Horten, Norway) with a 2.5-MHz transducer and an Aplio CV Toshiba system with a 3-MHz transducer, according to the American Society of Echocardiography guidelines (14). Left ventricular (LV) end-diastolic and end-systolic diameters, as well as wall thickness, were assessed using M-mode. LV ejection fraction and fractional shortening were measured in biplane 2D mode using Simpson's method. LV mass was estimated using the Devereux formula and normalized by height (in meters) (LVMi) raised to the 2.7 power to avoid underestimation in overweight or obese patients. Left ventricular hypertrophy (LVH) was determined as detailed in the Supplemental Methods, defined as LV mass/height ≥50 g/m^2.7^ for men and ≥47 g/m^2.7^ for women, following recent recommendations. LV geometry was examined using the four-tiered classification of LVH based on concentricity (defined as relative wall thickness ≥0.42) and LV end-diastolic volume (considered increased when LV end-diastolic volume/body surface area ≥74 mL/m^2^ in men and 61 mL/m^2^ in women). The intra-observer variation coefficient of echocardiography parameters was ≈4% for M-mode measurements and within 10% for 2D and Doppler-derived variables. Bland–Altman plots were used to verify the reproducibility of echocardiographic measurements and exclude systematic biases.

### Chronic kidney disease (CKD) assessment

Chronic renal disease was defined as an estimated glomerular filtration rate (eGFR) <60 mL/min/1.73 m^2^, calculated using the CKD-EPI formula, or persistently elevated 24-h albumin urinary excretion (15).

### Statistical analysis

All data are presented as mean ± standard deviation. Differences between means were assessed using Student's *t*-test or the Mann–Whitney *U*-test for non-normally distributed data in two-sample comparisons and by one-way analysis of variance with the Fisher least significant difference *post hoc* test for multiple comparisons. *χ*^2^ statistics were used to assess differences between categorical variables. Relationships between continuous variables were assessed by calculating the Pearson correlation coefficient or the Spearman rank correlation coefficient, as appropriate. Univariable logistic regression analysis was performed to evaluate the association between NLR and each established endpoint. Furthermore, we compared the predictive performance of NLR, fasting plasma glucose, creatinine, CRP, and uric acid using receiver operating characteristic (ROC) curves, with the area under the curve (AUC) estimated as a continuous variable for each marker. All tests were two-tailed, and analyses were performed using SPSS, version 25.0 (IBM). *p* values <0.05 were considered statistically significant.

## Results

**Figure 1:**
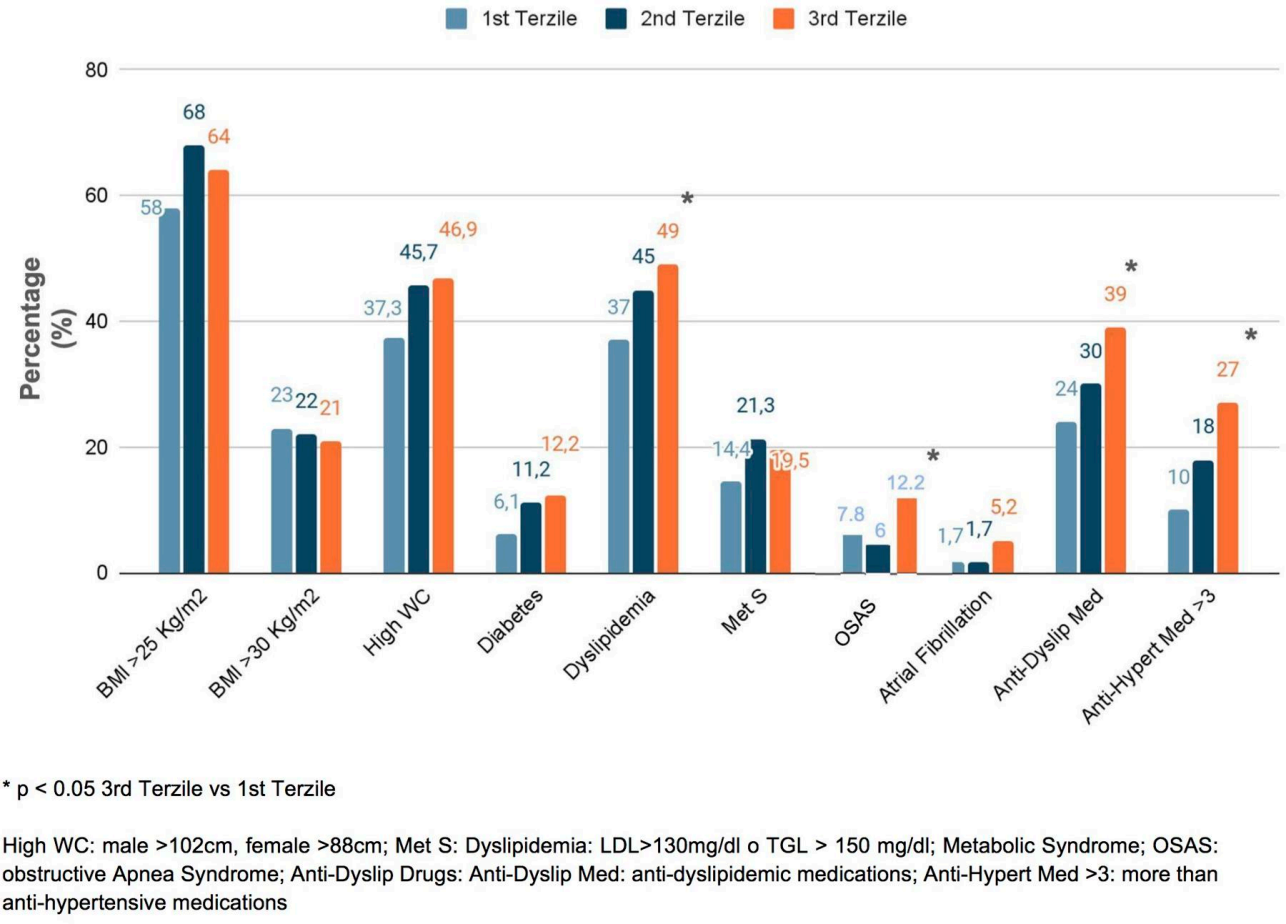
Prevalence of comorbidities in the enrolled patients, distinguished into tertiles of NLR.

In the study, we enrolled 346 consecutive patients with essential hypertension (mean age 53.4 ± 14.8 years; 46% women) (Table 1). In our cohort, the mean BMI was 26.8 ± 4.8 kg/m^2^, with mean SBP and DBP values of 141 ± 20 mmHg and 87 ± 12 mmHg, respectively. When stratifying the population into tertiles of NLR, we observed that patients in the third tertile were older (50.1 ± 15.7 vs. 58.7 ± 13.3 years, respectively; *p* < 0.001) and exhibited higher SBP (136 ± 18 vs. 143 ± 20 mmHg, *p* < 0.02) and DBP (85 ± 12 vs. 86 ± 12 mmHg, *p* < 0.01). No significant differences were observed for sex, BMI, and heart rate (HR) in all groups. Hypertensive patients in the third NLR tertile showed significantly higher serum creatinine levels (0.92 ± 0.16 vs. 0.99 ± 0.34 mg/dL, *p* = 0.04), while no significant differences were found in glucose blood levels or serum levels of low-density lipoprotein (LDL) and high-density lipoprotein (HDL) cholesterol (Table 2). Regarding comorbidities, patients in the third NLR tertile were more frequently affected by dyslipidemia (49%) and OSAS (12.2%) and were treated with more than three antihypertensive medications (27%) compared to those in the first NLR tertile (37%, 7.8%, and 10%, respectively; *p* < 0.05) (Figure 1).

**Table 1:** Anthropometric measurements in enrolled patients, distinguished by NLR tertiles.

Enrolled patients	Age	M/F	BMI	SBP	DBP	HR
	(years)	(%)	(kg/m^2^)	(mmHg)	(mmHg)	(bpm)
All patients (*n*=346)	53.4 ± 14.8	54/46	26.8 ± 4.8	141 ± 20	87 ± 12	68 ± 11
First tertile NLR (*n*=115)	50.1 ± 15.7	47/53	26.6 ± 4.8	136 ± 18	85 ± 12	68 ± 11
Second tertile NLR (*n*=116)	51.2 ± 13.6	58/42	27.1 ± 5.3	143 ± 21	90 ± 13	67 ± 10
Third tertile NLR (*n*=115)	58.7 ± 13.3*	58/42	26.6 ± 4.2	143 ± 20*	86 ± 12*	70 ± 12
*p*-Value Third vs. first tertile	**<0.001**	n.s.	n.s.	**<0.02**	**<0.01**	n.s.

M/F, male/female ratio; BMI, body mass index; SBP, systolic blood pressure; DBP, diastolic blood pressure; HR, heart rate.

Bold values report statistically significant comparisons.

*3rd vs 1st tertile <0.04.

**Table 2:** Biochemical parameters of enrolled patients, distinguished by NLR tertiles.

Enrolled patients	Creatinine	Glycaemia	LDL-C	HDL-C	Trgls	Uric acid	Microalbuminuria	VGF	NLR
	(mg/dL)	(mg/dL)	(mg/dL)	(mg/dL)	(mg/dL)	(mg/dL)	(mg/dL)	(mL/min/ 1.73 m^2^)	
All patients (*n*=346)	0.96 ± 0.26	94.3 ± 7.2	105.5 ± 13.3	51.6 ± 14.3	103 ± 20	5.61 ± 0.46	47.9 ± 9.3	81.6 ± 13.6	2.4 ± 1.2
First tertile NLR (*n*=115)	0.92 ± 0.16	93.9 ± 10.6	109.4 ± 18.8	52.1 ± 13.9	106.6 ± 29	5.68 ± 0.60	13.3 ± 3	84.6 ± 17	1.4 ± 0.2
Second tertile NLR (*n*=116)	0.96 ± 0.22	92.3 ± 9.8	101.8 ± 14	49.5 ± 12.8	104.6 ± 22	5.75 ± 0.43	45.3 ± 22	81.5 ± 13	2.2 ± 0.2
Third tertile NLR (*n*=115)	0.99 ± 0.34*	96.5 ± 11.6	105.5 ± 16.4	53.2 ± 15.8	96.8 ± 2	5.61 ± 0.47	87.3 ± 3*	78.7 ± 11*	3.6 ± 0.6*
*p*-value Third vs. first tertile	**0.04**	n.s.	n.s.	n.s.	n.s.	n.s.	**0.05**	**0.05**	**<0.001**

LDL-C, low-density lipoprotein-cholesterol; HDL-C, high-density lipoprotein-cholesterol; Trgls, triglycerides; VGF, velocity glomerular filtration.

Bold values report statistically significant comparisons.

*3rd vs 1st tertile <0.04.

**Figure 2:**
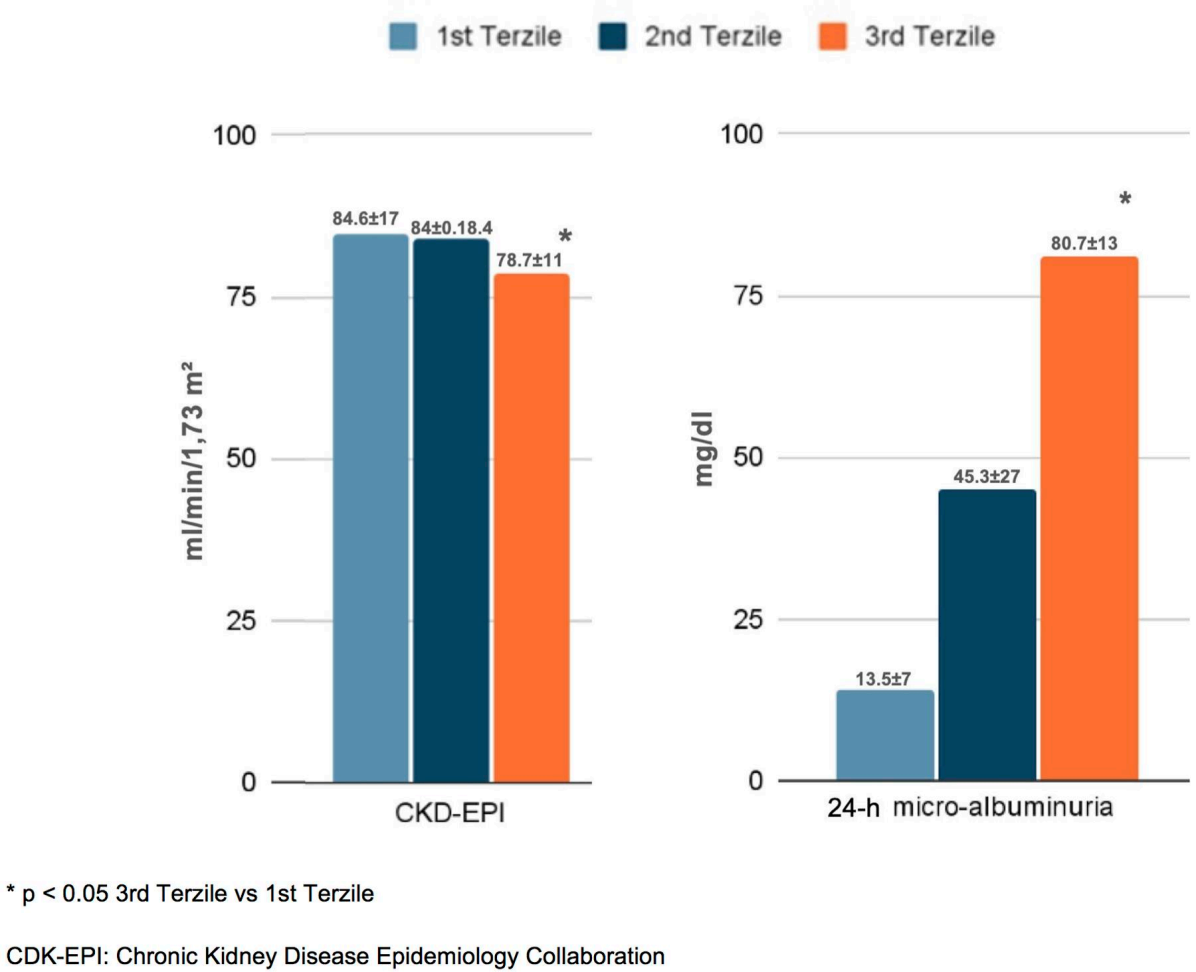
Cardiac and vascular damage in the enrolled patients, distinguished into tertiles of NLR.

**Figure 3:**
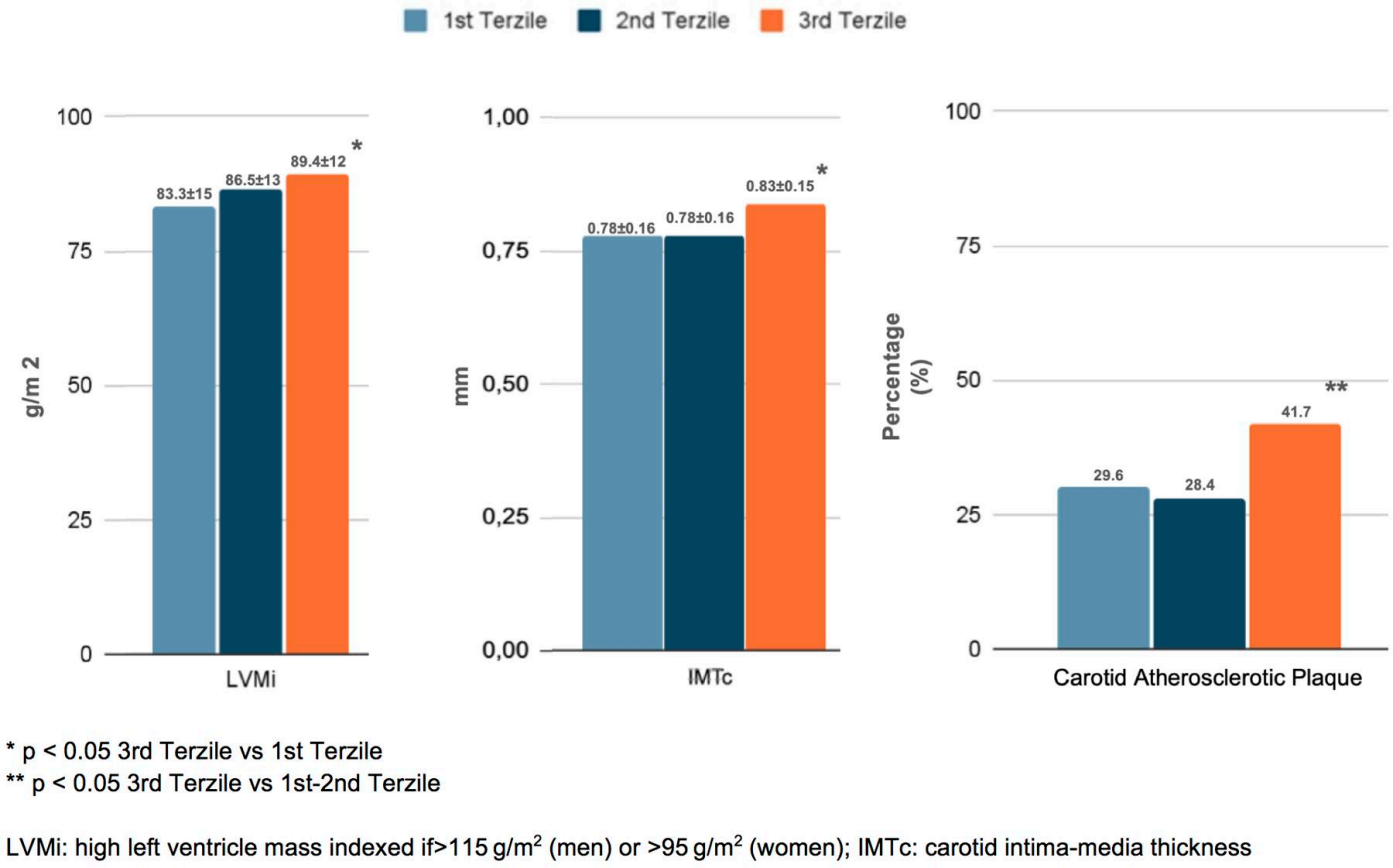
Renal damage in the enrolled patients, distinguished into tertiles of NLR.

In terms of organ damage, patients with higher NLRs exhibited a greater prevalence of subclinical cardiovascular changes. Specifically, those in the third tertile had a higher left ventricular mass index (LVMi) (89.4 ± 12 g/m²) and carotid intima–media thickness (cIMT) (0.83 ± 0.15 mm) compared to the first tertile (83.3 ± 14 g/m² and 0.78 ± 0.16 mm, respectively; *p* < 0.05). Additionally, carotid plaques were more frequent in the third tertile (41.7%) than in the first and second tertiles (29.6% and 28.4%, respectively; *p* < 0.05) (Figure 2). Furthermore, patients in the third NLR tertile showed significantly reduced kidney function, as indicated by a lower eGFR (78.7 ± 11 mL/min/1.73 m^2^) and higher 24-h urinary excretion of microalbuminuria (80.7 ± 13 mg/dL) compared to patients in the first NLR tertile (84.6 ± 17 mL/min/1.73 m^2^ and 13.5 ± 7 mg/dL, respectively; *p* < 0.05) (Figure 3).

We then conducted a univariable regression analysis to assess the association between NLR and study parameters. A higher NLR was strongly associated with increased LVMi [odds ratio (OR) 1.432, 95% confidence interval (95% CI) 1.080–1.896, *p* = 0.012], eGFR < 60 mL/min (OR 1.283, 95% CI 1.029–1.599, *p* = 0.027), cIMT > 0.9 mm (OR 1.266, 95% CI 1.040–1.543, *p* = 0.019), and the presence of carotid plaques (OR 1.307, 95% CI 1.072–1.594, *p* = 0.011) (Table 3).

**Table 3:** Univariate analysis of the association between NLR, target organ damage, and cardiovascular risk assessment.

Endpoint	Odds ratio	95% CI	*p*-Value
LVMi high	1.432	1.080–1.896	0.012
eGFR < 60 mL/min	1.283	1.029–1.599	0.027
cIMT > 0.9 mm	1.266	1.040–1.543	0.019
Carotid atherosclerosis	1.307	1.072–1.594	0.011
CVD risk	1.369	1.081–1.733	0.009

CVD risk: at least one cardiovascular risk factor (smoking, dyslipidemia, hypertension, diabetes mellitus, obesity).

**Figure 4:**
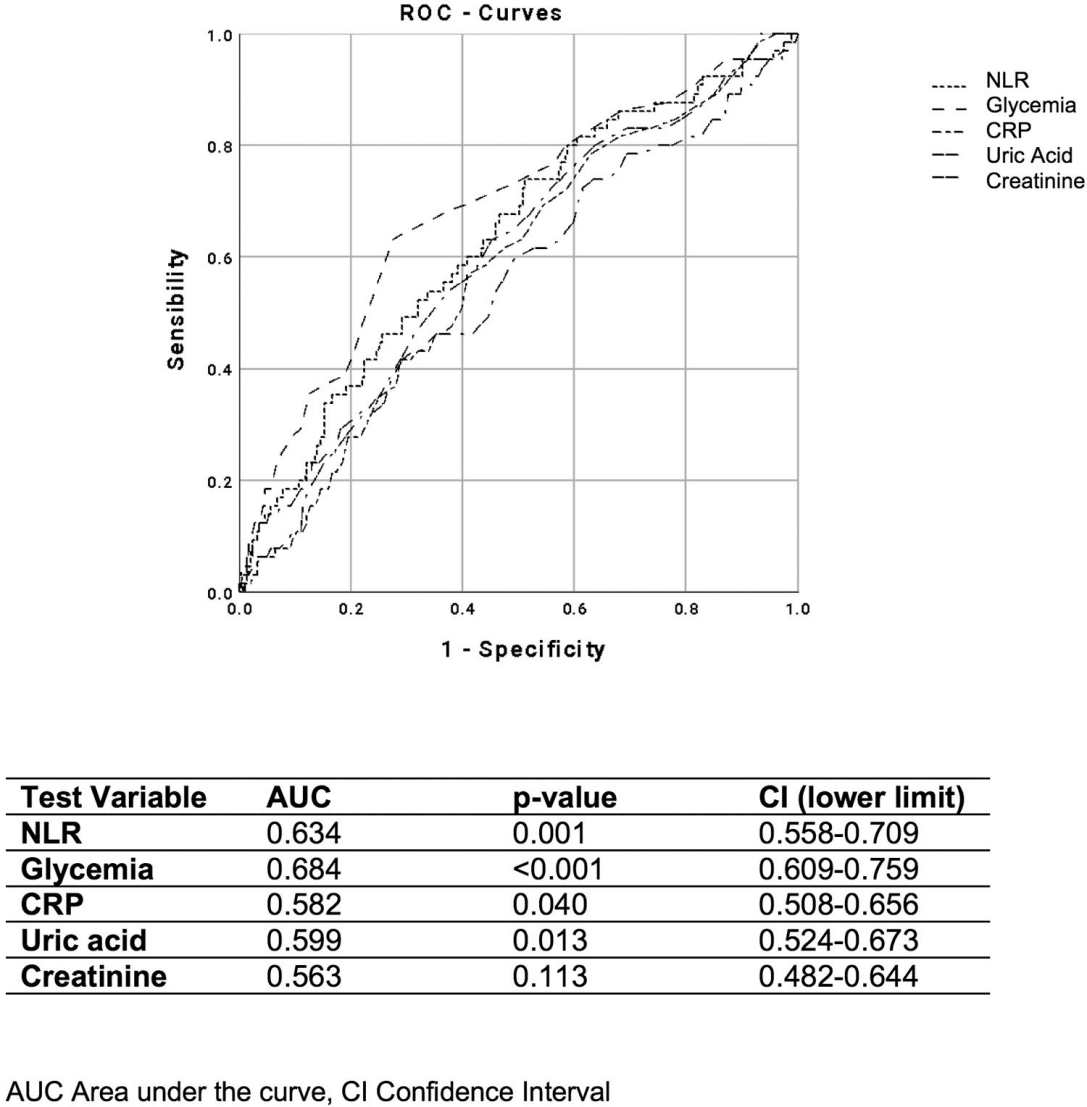
ROC curves of NLR, glycemia, CRP, plasma uric acid, and creatinine, for distinguishing the presence of two cardiovascular risk factors.

**Figure 5:**
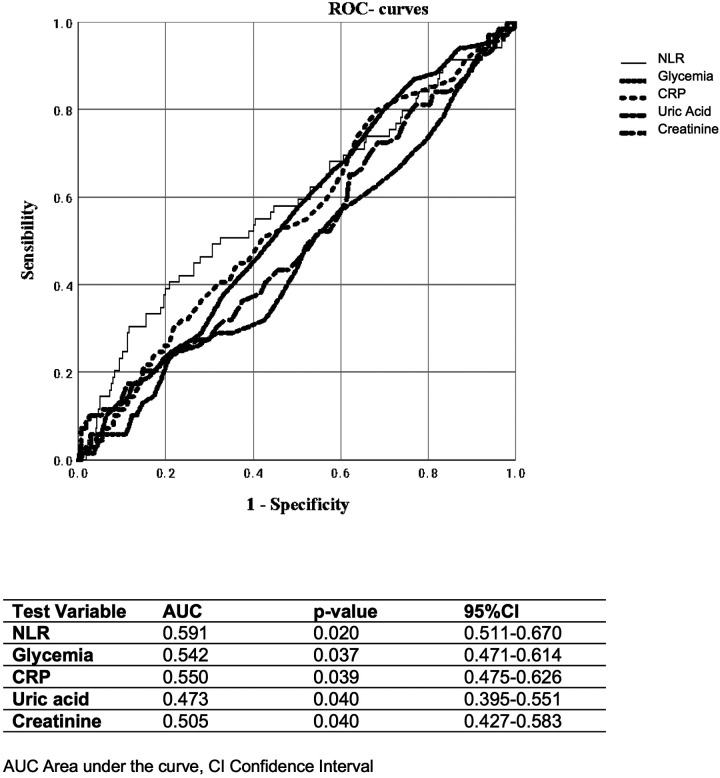
ROC curves of NLR, glycemia, C-reactive protein (CRP), plasma uric acid, and creatinine, for distinguishing patients with ≥3 antihypertensive drugs.

Finally, when evaluating different parameters, including glycemia, CRP, plasma uric acid, and creatinine, we found that NLR showed a stronger ability to identify the coexistence of at least two cardiovascular risk factors (AUC 0.591; *p* = 0.020) (Figure 4) and patients taking ≥3 antihypertensive medications or those with the presence of atherosclerotic plaques (AUC 0.634; *p* = 0.001) (Figure 5).

## Discussion

The NLR is a readily available and inexpensive index calculated from blood routine examinations and is considered a novel inflammatory biomarker that reflects two complementary immune pathways: the adaptive immune response mediated by lymphocytes (16) and the innate immune response mediated by neutrophils, which are responsible for non-specific inflammation reactions (7).

Several researchers have explored the clinical utility of NLR in assessing atherosclerotic complications, including carotid atherosclerotic plaques (17), mixed and non-calcified plaques in the coronary arteries (18), and the severity of coronary atherosclerosis (19). A systematic review reported that a high NLR was significantly associated with the risk of CAD (OR 1.62), ACS (OR 1.64), stroke (OR 2.36), and composite cardiovascular events (OR 3.86), underscoring its potential as a marker of CVD complications (20).

Furthermore, NLR has been significantly associated with several cardiometabolic conditions, including a “non-dipping pattern” in hypertensive patients (21–23), metabolic syndrome (24), and the coexistence of comorbidities such as heart disease, cancer, and diabetes (25). The pathophysiological relationship between NLR and atherosclerotic events is confirmed by the results of different intervention studies on large populations, such as the CANTOS, JUPITER, SPIRE-1/2, and CIRT trials (26). Data from the Rotterdam Study (2002–2014) showed that NLR levels were higher in men, older individuals, smokers, and those with lower socioeconomic status, diabetes, a history of cancer, or previous CVDs. After multivariate analysis, elevated NLRs were independently and significantly associated with an increased risk of all-cause mortality (HR 1.64) and cardiovascular mortality (HR 1.92) (27).

In this study conducted on patients with essential hypertensive without a history of CVD events, we observed that elevated NLRs were associated with several comorbidities and subclinical atherosclerotic complications. Specifically, higher NLR was associated with elevated arterial blood pressure, a higher prevalence of patients treated with more than three antihypertensive medications, and a higher prevalence of dyslipidemia. Atherosclerosis is a degenerative process characterized by enhanced chronic inflammation; in this regard, NLR is closely related to the chronic inflammatory state. NLR is involved in the regulation of arterial function and the progression of atherosclerosis through interactions with the endothelium, platelets, and neutrophil infiltration (28, 29).

In this research, we observed a higher prevalence of visceral obesity and OSAS in patients with elevated NLR, both conditions characterized by enhanced chronic inflammation. Visceral adiposity is closely related to subclinical inflammation and CVDs. As regards, Bagyura et al. evaluated the association between NLR and coronary artery calcium score (CACS), finding a close interaction between tertiles of visceral adiposity, NLR, and CACS (30, 31). Regarding OSAS, Uygur et al. investigated the association between NLR and the severity of OSAS, finding significant associations between NLR and apnea–hypopnea index (*r*: 0.448), mean SaO_2_ (*r*: 0.341), and oxygen desaturation index (*r*: 0.327) (32). Several studies have shown that levels of inflammatory markers, including CRP, IL-6, and tumor necrosis factor, are elevated in patients with OSAS (33). It is well established that endothelial dysfunction caused by inflammatory processes plays a key role in the development of coronary artery disease, atherosclerosis, and other cardiovascular complications in patients with OSAS (34). Dysregulation of neutrophil apoptosis and increased expression of adhesion molecules may contribute significantly to the atherosclerotic process of OSAS (35).

While previous studies have demonstrated an association between NLR and the development of major atherosclerotic complications (i.e., coronary arteriopathy or peripheral arteriopathy), our study focused on patients with essential hypertensive without previous CVD events. We found that elevated NLRs were significantly correlated with a higher prevalence of cardiovascular remodeling [LVMi, cIMT, and atherosclerotic plaques] and chronic kidney disease (reduced glomerular filtration and increased microalbuminuria).

cIMT is a widely recognized method for CVD risk stratification. NLR has been evaluated as an independent risk factor for the development of asymptomatic atherosclerosis in populations with or without DM. In prediabetic and diabetic groups, studies conducted by Lee and Li compared patients with normal cIMT (cIMT <0.9 mm) to those with elevated cIMT (≥1 mm), finding that the latter group had higher mean NLR values, which were significantly correlated with age, HbA1c, and systolic blood pressure (36, 37). Several studies have reported increased levels of pro-inflammatory cytokines in prediabetic and diabetic patients (38), finding that persistent hyperglycemia continuously activates neutrophils, resulting in infiltration and damage of vascular endothelial cells (39). In contrast, lymphocytopenia is considered an inflammatory marker, particularly in conditions with increased corticosteroid levels in response to stress, which are associated with increased inflammatory reactions and lymphocyte apoptosis (40).

The relationship between type 2 DM and chronic inflammatory state is bidirectional. Type 2 DM is a itself chronic inflammatory condition, characterized by increased differentiation of monocytes into macrophages (41); however, on the other hand, the chronic inflammatory state promotes insulin resistance and the development of type 2 DM through altered signaling of inflammatory molecules (i.e., IL-6) in the liver (41).

In our study, increased cardiac remodeling and vascular damage were associated with higher pressure overload, which correlated significantly with NLR levels. Arterial hypertension is characterized by a progressive inflammatory process, involving the accumulation of innate and adaptive immune cells at vascular level and in the interstitium of affected organs (4). NLRs above 2.7 have been related to greater blood pressure variability and a more frequent non-dipping pattern, suggesting that this marker is an indicator of increased risk of related adverse cardiovascular events in hypertensive patients (42, 43). In a study on patients in secondary prevention, NLR, BNP, and CRP levels were higher in eccentric and concentric LVH compared to those without LVH (44). Other studies on asymptomatic patients have highlighted a higher prevalence of subclinical organ damage. Karagöz et al. reported greater diastolic dysfunction in patients with elevated NLRs (45), while studies on pediatric subjects with essential hypertension have shown that NLR is a useful marker of arterial damage/stiffness, correlating with diastolic, systolic, and mean blood pressure, as well as with PWV (46).

A limitation of the present study is the absence of additional biochemical markers of chronic inflammation (i.e., IL-6 and other cytokines) and specific subclasses of inflammatory cells, which could be potentially explored in future research.

In conclusion, this present study shows that in patients affected by arterial hypertension, evaluated in primary prevention, the NLR—an easily obtainable, repeatable, and low-cost marker—is significantly correlated with a higher prevalence of several comorbidities and elevated blood pressure. Moreover, it has a predictive ability for subclinical organ damage at the cardiac, vascular, and renal levels, making it a valuable surrogate marker of atherosclerotic damage.

## Data Availability

The raw data supporting the conclusions of this article will be made available by the authors, without undue reservation.
